# Characteristics of the transgender community in Lebanon and barriers to proper healthcare

**DOI:** 10.3389/fgwh.2026.1828265

**Published:** 2026-07-17

**Authors:** Diala Harkous, Mariana Boulos, Marie-Hélène Gannagé-Yared, Nada El Ghorayeb

**Affiliations:** Department of Endocrinology at Hotel-Dieu de France Hospital, Faculty of Medicine, Saint-Joseph University, Beirut, Lebanon

**Keywords:** discrimination, gender-affirming care, HIV, Lebanon, lesbian, gay, bisexual, transgender and queer (LGBTQ), mental health, minority stress, transgender health

## Abstract

**Background:**

Individuals with gender dysphoria represent a growing portion of the global population. Yet data about this community is lacking in Lebanon.

**Objectives:**

This study aimed to characterize the health status of the transgender and gender-diverse (TGD) Lebanese adults by addressing challenges and disparities in access to basic needs.

**Methods:**

An anonymous online survey was conducted between January and May 2021 across the Non Governmental Organization (NGO) supporting TGD individuals in Lebanon. The study consisted of self-reported health assessments collecting data on medical and mental disorders, social stigma and discrimination, gender-affirming therapies, and barriers to proper healthcare.

**Results:**

Among forty-eight participants, 75% were assigned as male sex at birth (AMAB), and 25% as female (AFAB). Median age was 30 years. Approximately half (58.3%) of the cohort was unemployed despite 81.2% having completed high school or a university degree. Non-medical gender affirmation was observed in 79.2%. Gender-affirming hormone therapy was used in 52.8% of AMAB and in 58.3% of AFAB gender-affirming surgical procedures were done in 30.5% of AMAB and in 50% of AFAB. TGD individuals tested positive for HIV in 14.6% of the cases. The use of illicit drugs was reported in 35.4%. Mental health problems were found in 83.3% of AMAB and 91.7% of AFAB. They claimed being victims of assault in 83.3%, and faced discrimination in 87.5%. Barriers to access to medical care were observed in 75%, mainly due to financial limitations (47.2%) and lack of medical expertise in 10.4%.

**Conclusion:**

In Lebanon, TGD individuals still struggle for basic services due to rigid sociocultural norms and the ongoing economic crisis. There is an urgent need to adopt health policies and train healthcare providers in order to provide a safe and appropriate gender affirmation to these individuals.

## Introduction

Gender and sexuality are complex aspects of individuals' identities (1). By definition, the terms “transgender and gender diverse” (TGD) describe an umbrella including all people whose gender identity and/or gender expression differs from what is typically associated with their sex assigned at birth (2). The proportion of individuals with diverse sexual orientation, gender identity and expression, and sex characteristics (SOGIESC) is increasing worlwide reaching around 0.4% to 0.7% of the adult population (3). According to a large European survey of 18,600 transgender participants, approximately 54% were assigned female at birth (AFAB), and 46% were assigned male at birth (AMAB) (4). TGD individuals experience high levels of violence and discrimination, which negatively affect their mental health and wellbeing. Common challenges include stigma, social stress, peer victimization, and family rejection (5–10). In Lebanon, TGD individuals face discrimination driven by a conservative legal climate and social attitudes. Despite increasing public visibility of gender-diverse individuals in Lebanon, stigma and discrimination remain significant. Characterizing the sociodemographic and health-related needs of TGD individuals is therefore critical for the development of inclusive public health policies. Despite advances in gender-specific health research, important gaps remain in the understanding of TGD healthcare, largely due to the limited availability of community-based longitudinal data (10), especially, in the Middle East and North Africa (MENA) region.

To our knowledge, no community-based study has comprehensively characterized TGD individuals in Lebanon. This survey aimed to assess demographic characteristics, gender identity and expression, psychiatric and medical conditions, health risk behaviors, and use of gender-affirming interventions, while examining healthcare disparities and unmet needs. The results are intended to inform efforts to enhance equitable healthcare access and delivery.

## Methods

### Participant recruitment and survey delivery

A questionnaire-based study was conducted between January and May 2021, in the Arabic language and administered to eligible participants, by email or by a URL link shared on the WhatsApp Application via Google Docs format to limit social desirability bias that can challenge some of the individuals. Participation in the survey was voluntary and fully anonymous. While the recruitment process utilized elements of respondent-driven sampling (RDS) facilitated by the local LGBTQ non-governmental organization (NGO) “PROUD LEBANON,” several parameters of a formal RDS design were omitted due to the hard-to-reach nature of this population. Initial “seeds” were purposively selected from individuals actively accessing legal, healthcare, or psychosocial support services at PROUD LEBANON. These seeds were provided with unique URL links to share via WhatsApp within their social networks. However, the exact number of successive recruitment waves was not formally tracked, and a fixed coupon-rationing system was not enforced. To minimize duplicate responses while preserving strict anonymity, the survey system restricted submissions to one entry per IP address/device session, and the inclusion criteria explicitly required participants to confirm they were taking the survey for the first time. No statistical weighting (e.g., RDS-II/Volz-Heckathorn estimators) was applied to adjust for network size or homophily; data are analyzed as unweighted sample proportions.

All data collection forms were securely stored, with access limited to the researchers only. The inclusion criteria were defined as follows: self-identification as a TGD person, aged over 18 years, capable of reading and understanding Arabic, residing in Lebanon, participating in the survey for the first time, and willing to provide written informed consent.

### Questionnaire content

The questionnaire collected demographic information, including age, sex assigned at birth, and gender identity, using predefined categories (male, female, both male and female, neither male nor female). Participants were asked about psychiatric conditions [depression, anxiety, post-traumatic stress disorder (PTSD), personality disorders, eating disorders, and schizophrenia] and psychiatric events such as suicidal ideation, attempts, self-injury, and hospitalizations.

Simple and concise questions were used rather than utilizing validated psychometric screening tools (such as the PHQ-9 for depression, GAD-7 for anxiety, or PCL-5 for PTSD), because it could be very difficult for those individuals to answer without assistance.

Medical history was documented across a broad range of conditions, including: obesity, hypertension, hypercholesterolemia, hypertriglyceridemia, impaired fasting glucose or prediabetes, type 1 diabetes, type 2 diabetes, pulmonary embolism, deep venous thrombosis, cerebrovascular accident, myocardial infarction, osteoporosis, osteopenia, fracture, cancer, human immunodeficiency virus (HIV), and non-HIV sexual transmitted infections (STIs). Health-risk behaviors were assessed, including experiences of abuse and substance use. The questionnaire also included questions related to experience of bullying, discrimination, and barriers to services (healthcare, employment, religious institutions, restaurants/bars, and housing), as well as sources of support from family, friends, Lesbian, Gay, Bisexual, and Transgender and Queer (LGBTQ) community centers, support groups, and chat lines. Finally, gender-affirming medical and surgical treatments were recorded, as well as access to behavioral health evaluations and ongoing counseling.

### Ethical considerations

This study was conducted in accordance with the principles of the Declaration of Helsinki. The project was approved by the Ethics committee of the Hotel-Dieu de France hospital, Lebanon (reference CEHDF 1992).

### Informed consent

A written informed consent was obtained from all participants prior to participation.

### Statistical methods

No formal *a priori* sample size or statistical power calculation was performed for this study. Given the small sample size (*N* = 48) and recruitment through a single localized community center, it was considered as an exploratory convenience study.

Statistical Package for the Social Sciences (SPSS) (IBM corp. IBM SPSS Statistics for Windows, version 26.1. Armonk,NY) was used for data analysis, Continuous variables were presented as means ± SD or median (IQR), and categorical variables as counts and percentages. Comparisons between groups were assessed with the Mann–Whitney test for continuous variables. To compare categorical characteristics and prevalence rates between individuals assigned male at birth (AMAB) and those assigned female at birth (AFAB), Fisher's exact test was utilized due to the small expected cell counts (frequently <5) across the cross-tabulations; standard Chi-square tests were used where cell count assumptions were met. Significance level had been set at 5% (*p*-value less than 0.05).

## Results

### Study population characteristics

A total of forty-eight TGD adults filled out the questionnaire. The median age was 30 years (26–39), with 62.5% being under the age of 35. Seventy-five percent were AMAB, and twenty-five percent AFAB. Among AMAB individuals, 58.4% self-identified as female and 19.4% as genderqueer, while among AFAB individuals, 75% expressed themselves as male and 8.4% as genderqueer. Most of them (90%) were residing in the capital city, Beirut, and in Mount Lebanon. Approximately half of the cohort (58.3%) was unemployed despite having a high school degree in 39.6%, or a university diploma in 41.7%. Non-medical gender affirmation was achieved in 79.2% of the participants. In addition, 52.8% of AMAB and 58.3% of AFAB were receiving gender- affirming hormone therapy. Finally, 30.5% of AMAB and 50% of AFAB subjects had undergone at least one gender-affirming surgical procedure.

### Reported mental health and physical disorders

Mental Health problems were prevalent with over 85% of TGD participants experiencing depression, anxiety, or PTSD (91.7% of AFAB and 83.3% of AMAB). Suicide attempt, suicide Ideation and substance use were reported in 12.5%, 39.6% and 35.4% of TGD individuals respectively (Table 1). At least one chronic medical condition was identified in 41.7% of TGD participants. HIV infection being the most frequent one and reported in 14.6%. Low bone mass density was found in 6.3% only but metabolic syndrome and cardiovascular diseases were detected in 15% of the cohort (Table 1). There was no statistically significant difference in the prevalence of chronic conditions between AFAB and AMAB participants.

**Table 1:** Self reported medical and mental disorders in TGD individuals.

Medical and mental disorders	Total cohort *N* = 48 (%)	AMAB *N* = 36 (%)	AFAB *N* = 12 (%)	*P* value (AMAB vs. AMAB)
Any chronic medical disease	20 (41.7%)	18 (50%)	2 (16.7%)	***p* = 0.04**
HIV infection (confirmed)	7 (14.6%)	6 (16.7%)	1 (8.3%)	*p* = 0.48
Obesity	2 (4.2%)	1 (2.7%)	1 (8.3%)	*p* = 0.40
Low bone density	3 (6.3%)	3 (8.3%)	0 (0%)	*p* = 0.30
Diabetes	1 (2.1%)	1 (2.7%)	0 (0%)	*p* = 0.56
Dyslipidemia	1 (2.1%)	1 (2.7%)	0 (0%)	*p* = 0.56
Cerebral Vascular Accident	1 (2.1%)	1 (2.7%)	0 (0%)	*p* = 0.56
Cancer	1 (2.1%)	1 (2.7%)	0 (0%)	*p* = 0.56
Other medical conditions	4 (8.3%)	4 (11.1%)	0 (0%)	*p* = 0.23
Any illicit drug use (past 12 months)	17 (35.4%)	11 (30.5%)	6 (50%)	*p* = 0.2
Any psychiatric diagnosis (self-report/clinician)	42 (85%)	31 (86.1%)	11 (91.7%)	*p* = 0.6
Suicide attempt	6 (12.5)	3 (8.3%)	3 (25%)	*p* = 0.13
Suicide ideation	19 (39.6%)	14 (38.8%)	5 (41.7%)	*p* = 0.86

Data are presented as percentages. Significant *p* values are shown in bold.

AFAB, assigned female at birth; AMAB, assigned male at birth; TGD, transgender and gender diverse.

### Violence and discrimination against TGD individuals

Experiences of victimization and structural marginalization were pervasive across the cohort, with 83.3% of the total population reporting being a victim of some form of assault, and 87.5 reporting experiencing at least one form of discrimination (Table 2). For instance, verbal assault was experienced by 27.1% of the total cohort, showing a higher descriptive but non-significant trend in AFAB (41.7%) compared to AMAB individuals (22.2%; *p* = 0.19). Physical assault affected 39.6% of the total sample, distributed almost equally between AMAB (38.8%) and AFAB individuals (41.7%; *p* = 0.875). Furthermore, sexual assault was reported by 16.7% of the total cohort; notably, all reported victims belonged to the AMAB group (22.2% vs. 0% in AFAB), trending toward significance (*p* = 0.07). Structural and institutional discrimination were highly prevalent, particularly in economic spheres (Table 2). In fact, half of the entire cohort (50.0%) reported facing overt discrimination in employment, representing the most common structural barrier (58.3% of AFAB vs. 47.2% of AMAB; *p* = 0.50).

**Table 2:** Self reported acts of violence or discrimination against TGD individuals.

Acts of violence or discrimination	Total cohort *N* = 48 (%)	AMAB *N* = 36 (%)	AFAB *N* = 12 (%)	*P* value (AMAB vs. AMAB
Victim of verbal assault	13 (27.08%)	8 (22.2%)	5 (41.7%)	*p* = 0.19
Victim of physical assault	19 (39.58%)	14 (38.8%)	5 (41.7%)	*p* = 0.875
Victim of sexual assault	8 (16.67%)	8 (22.2%)	0 (0%)	*p* = 0.07
Discrimination in health care	8 (16.67%)	7 (19.4%)	1 (8.3%)	*p* = 0.37
Discrimination in housing	6 (12.5%)	6 (16.6%)	0 (0%)	*p* = 0.13
Discrimination in employment	24 (50%)	17 (47.2%)	7 (58.3%)	*p* = 0.50
Discrimination in religious beliefs	3 (6.25%)	3 (8.3%)	0 (0%)	*p* = 0.30

Data are presented as percentages.

AFAB, assigned female at birth; AMAB, assigned male at birth; TGD, transgender and gender diverse.

Likewise, overt discrimination within healthcare settings was reported by 16.7% of the total cohort (19.4% AMAB vs. 8.3% AFAB; *p* = 0.37). Finally, discrimination in housing was reported by 12.5% of the sample (isolated entirely to AMAB individuals at 16.6%; *p* = 0.13), while 6.3% reported discrimination based on religious beliefs (8.3% AMAB vs. 0% AFAB; *p* = 0.30).

### Barriers to healthcare

The majority (75%) were facing challenges to obtain access to medical care, whether in private clinics or hospitals. These include mainly financial constraints in 47.2% of the cases. Fear of stigma among health care workers due to limited nurses and physicians having expertise in dealing with TGD individuals was reported in 12.5%. Availability of gender-affirming medications was also a barrier due to scarcity of medications during the study period when the country was facing a huge economic crisis. Fear of side effects from gender-affirming therapy was also observed in 12.5% due to poor knowledge or awareness of the benefits vs. dangers of such therapies (Table 3). There were no statistically significant differences between AFAB and AMAB participants with respect to barriers to healthcare.

**Table 3:** Barriers to healthcare access in TGD individuals.

Barriers	Total cohort *N* = 48 (%)	AMAB *N* = 36 (%)	AFAB *N* = 12 (%)	*P* value (AMAB vs. AMAB)
Reported any barrier to accessing gender-affirming care Medications	5 (10.42%)	3 (8.3%)	2 (16.7%)	*p* = 0.41
Financial cost/inability to pay	17 (35.42%)	13 (36.1%)	4 (33.3%)	*p* = 0.86
Fear of side effets	6 (12.5%)	5 (13.9%)	1 (8.3%)	*p* = 0.61
Lack of knowledgeable M.D. providers	3 (6.25%)	1 (2.8%)	2 (16.7%)	*p* = 0.09
Ever self-administered hormones without medical supervision due to lack of MD	5 (10.41%)	4 (11.1%)	1 (8.3%)	*p* = 0.79
No Barrier	12 (25%)	10 (27.8%)	2 (16.7%)	*p* = 0.44

Data are presented as percentages.

AFAB, assigned female at birth; AMAB, assigned male at birth; TGD, transgender and gender diverse.

## Discussion

This study provides one of the first description of the characteristics of the Lebanese TGD community. Similarly to the European national survey (11) and to a recent stratified analysis from Italy, our results highlight substantial disparities in mental and physical health, socioeconomic status, and access to healthcare among TGD individuals (12, 13).

### Socioeconomic Status

Despite a highly educated sample where 81.3% completed high school or attained a university degree, the cohort faces a disproportionate unemployment rate of 58.3%. This profound disconnect mirrors patterns seen in Western datasets like the U.S. Transgender Survey (USTS) (12–14). This demonstrates that educational attainment does not insulate TGD individuals from economic marginalization. In Lebanon, this is severely exacerbated by the complete absence of workplace anti-discrimination protections and legal pathways for gender recognition. Without matching legal gender markers on identification documents, navigating job applications, contract signings, and interviews exposes individuals to systemic structural exclusion and hiring biases.

### Mental health

The self-reported prevalence of mental health struggles (85% overall; 91.7% of AFAB and 86.1% of AMAB) is remarkably elevated compared to European data (12, 13), in which over half of participants reported depression and/or anxiety. These outcomes map onto classic Minority Stress Theory, which posits that chronic, hostile social environments create excess psychological burdens (15–17). Echoing findings from Italian and European surveys, non-binary participants and AFAB individuals in our sample reported high rates of psychiatric comorbidity and poorer health outcomes. However, the Lebanese context amplifies these disparities due to more pronounced structural discrimination. The very high levels of exposure to physical or sexual assault (83.3%) and discrimination (87.5%) are consistent with findings from other Lebanese studies documenting pervasive transphobia and violence (18–20). In these studies, 49% of participants reported a history of psychological trauma, 68% experienced physical violence and 98% reported discrimination (18–20). Compared to European and North American estimates, where TGD participants experienced marginalization on around 25% of days over 56 days of daily surveys. Both daily experiences and long-term structural contexts of transphobia converge in contributing to adverse mental health outcomes (17). The high rate of composite suicidal ideation or attempt (56%) directly aligns with pervasive local proximal and distal stressors. This is consistent with other Lebanese studies (46%) (17, 20), but remains substantially higher than global pooled estimates (∼29%) (21). Because the study relies on simple self-report rather than clinical tools, these figures may represent acute psychological distress tied to immediate survival challenges within a hostile environment rather than baseline endogenous psychiatric disorders.

### Physical health and HIV

Physical health of TGD is threatened by a broader burden of chronic conditions (41.7% of participants reported living with at least one chronic medical condition). These findings are consistent with European cohort studies showing elevated burdens of physical illness among TGD adults (12, 13). Italian TGD adults report high rates of long-term health conditions (thyroid disease, osteoporosis, metabolic syndrome) and worse overall physical health compared to general population norms. Smoking and binge drinking are significantly more common in TGD adults than in the general Italian population, while uptake of preventive care (such as cancer screenings) is lower. Many TGD people avoid or delay medical care due to past discrimination, anticipated stigma, or providers' lack of transgender-competent training. Over 40% of surveyed TGD adults reported feeling discriminated against in healthcare settings (13). The documented HIV prevalence of 14.6% represents a severe concentrated public health issue, tracking significantly higher than general population estimates in Europe and previous regional data. HIV prevalence among transgender women being estimated at 17%–19% (9, 22). This concentration is best understood via a syndemic framework: economic marginalization (58.3% unemployment) and high rates of sexual and physical assault (Table 2) intersect, forcing individuals into survival economies or unstable housing situations that amplify exposure to HIV and other STIs. Furthermore**,** substance use was reported by 35.4%, comparable to global and European transgender studies (23). Minority stress and trauma histories likely contribute to these patterns, where mental illness, substance use, and HIV risk amplify one another (17).

### Access to gender-affirming care

A total of 75% of our participants reported facing barriers such as financial constraints (35.42% in Table 3), provider stigma, and the absence of knowledgeable healthcare expertise in gender-affirming services (6.25%). Comparable results are observed in Italian and European studies, although Lebanese barriers appear more severe, mainly due to limited trained providers, absence of structured gender clinics, and fragmented health care system (12, 24, 25). In our cohort, 52.8% of AMAB and 58.3% of AFAB were using gender-affirming hormone therapy, similarly to the Italian cohort (12, 13). Unfortunately, according to our results and other Lebanese data, many TGD individuals lack supervised medical care, contrary to Endocrine Society recommendations (2) which emphasize evidence-based endocrine protocols where these individuals should undergo follow-up visits and hormonal testing every 3 to 6 months (19, 26). Moreover, the World Professional Association for Transgender Health [WPATH] SOC-8 (27), Endocrine Society (2), highlight access to gender-affirming care as protective against depression, suicidality, and psychological distress (26, 28). In Lebanon, this is heavily compounded by the severe ongoing economic crisis, which has decimated drug supplies, making prescribed transaffirmative medications highly scarce and prohibitively expensive.

### Policy implications

Addressing these inequities requires structural, multi-sectoral interventions. Unlike many Western countries, Lebanon currently lacks legal gender recognition policies, anti-discrimination laws, insurance coverage, or public-sector gender clinics emphasizing the need for multiple concrete actions in the Lebanese legislation. First, is to implement standardized, accessible, gender-affirming care pathways. Second, the need for a Lebanese medical curriculum integrating standardized provider training on TGD health, to eliminate provider stigma and build clinical capacity. Furthermore, is the integration of mental-health, HIV with the primary-care basic services and to become accesible to all. Finally, long-term mitigation of minority stress demands legal reforms guaranteeing non-discrimination protections in employment and housing, alongside safe access to healthcare. The benefit of these implementations such as provider training was demonstrated in two Italian studies (12, 13). These steps are essential to reducing disparities and improving well-being among TGD individuals in Lebanon. Because poor outcomes are not inherent to transgender identity but are the predictable result of hostile environments and insufficient protections targeting these frameworks will probably improve overall well-being and safety.

### Nuancing the Selection Bias

Our study has several strengths. It is the first Lebanese study that provides unique, systematically collected data on health and social experiences of TGD individuals. In addition, the survey was conducted in collaboration with a trusted LGBTQ NGO, which increased participant trust and reduced fear of disclosure, therefore improving data validity. Furthermore, the study collected data across social, mental-health, physical-health, and healthcare-access domains, allowing for a multidimensional analysis grounded in established models such as minority-stress theory.

However, the study has some limitations. The sample size was small (*N* = 48) mainly because it is performed on a marginalized population, which might limit generalizability. A key limitation is the recruitment strategy, as participants were drawn from a single, specialized LGBTQ NGO in Beirut. This introduces a complex, dual-directional selection bias that must shape interpretation. Individuals often seek NGOs because participants have unique access, they have experienced acute violence, to legal aid, support groups, homelessness, or medical crises. and safer healthcare networks. As a result, the sample may overrepresent highly visible or service-engaged individuals while underrepresenting isolated TGD individuals who do not access NGO support. In addition, respondent-driven sampling, although useful, may bias recruitment toward individuals connected to NGOs or community networks. Psychiatric diagnoses, experiences of violence, substance use, and HIV status were self-reported and may be subject to recall or reporting bias. The Cross-sectional study cannot determine causality between discrimination, minority stressors, and health outcomes. AFAB individuals were underrepresented, limiting subgroup analyses. Biological markers, treatment histories, and detailed clinical data were not verified using medical records because of limited access to documentation. Finally, stigma and fear may have discouraged some individuals from participating, potentially leading to an underestimation of the prevalence of violence or HIV infection.

## Conclusion

This study provides foundational evidence on the lived experiences of TGD individuals in Lebanon. High levels of discrimination, violence, psychiatric morbidity, HIV prevalence, and barriers to gender-affirming care reflect a broader environment of structural stigma and inadequate health-system capacity. These findings align with global evidence demonstrating that minority stress, unmet medical needs, and systemic exclusion drive health inequities in transgender populations. Importantly, international guidelines, including those of the Endocrine Society and WPATH, consistently show that gender-affirming care improves mental-health outcomes, reduces suicidality, and enhances overall well-being. Lebanon urgently requires structural interventions: legal protections, provider education, expansion of gender-affirming services, and integration of mental-health, HIV, and primary care services in safe, nondiscriminatory environments. Strengthening national policies and investing in culturally sensitive, trauma-informed, gender-affirming care pathways are essential steps toward reducing disparities and supporting the health and dignity of TGD communities.

## Data Availability

The original contributions presented in the study are included in the article/Supplementary Material, further inquiries can be directed to the corresponding author.

## References

[1] Johnson B Leibowitz S Chavez A Herbert SE. Risk versus resiliency: addressing depression in lesbian, gay, bisexual, and transgender youth. Child Adolesc Psychiatr Clin N Am. (2019) 28(3):509–21. 10.1016/j.chc.2019.02.016 31076124

[2] Hembree WC Cohen-Kettenis PT Gooren L Hannema SE Meyer WJ Murad MH. Endocrine treatment of gender-dysphoric/gender-incongruent persons: an endocrine society* clinical practice guideline. J Clin Endocrinol Metab. (2017) 102(11):3869–903. 10.1210/jc.2017-01658 28945902

[3] Meerwijk EL Sevelius JM. Transgender population size in the United States: a meta-regression of population-based probability samples. Am J Public Health. (2017) 107(2):e1–8. 10.2105/AJPH.2016.303578 28075632 PMC5227946

[4] Vandendriessche C Cohen D. Social factors behind the AFAB predominance in LGBT youths: evidence from a large European survey. Eur Child Adolesc Psychiatry. (2025) 34(7):2093–106. 10.1007/s00787-024-02595-4 39625682 PMC12334450

[5] Müller A. Scrambling for access: availability, accessibility, acceptability and quality of healthcare for lesbian, gay, bisexual and transgender people in South Africa. BMC Int Health Hum Rights. (2017) 17(1):16. 10.1186/s12914-017-0124-4 28558693 PMC5450393

[6] Rees SN Crowe M Harris S. The lesbian, gay, bisexual and transgender communities’ mental health care needs and experiences of mental health services: an integrative review of qualitative studies. J Psychiatr Ment Health Nurs. (2021) 28(4):578–89. 10.1111/jpm.12720 33295065

[7] Hafeez H Zeshan M Tahir MA Jahan N Naveed S. Health care disparities among lesbian, gay, bisexual, and transgender youth: a literature review. Cureus. (2017) 9(4):e1184. 10.7759/cureus.1184 28638747 PMC5478215

[8] Winter S Diamond M Green J Karasic D Reed T Whittle S. Transgender people: health at the margins of society. Lancet. (2016) 388(10042):390–400. 10.1016/S0140-6736(16)00683-8 27323925

[9] Reisner SL Poteat T Keatley J Cabral M Mothopeng T Dunham E. Global health burden and needs of transgender populations: a review. Lancet. (2016) 388(10042):412–36. 10.1016/S0140-6736(16)00684-X 27323919 PMC7035595

[10] James HA Chang AY Imhof RL Sahoo A Montenegro MM Imhof NR. A community-based study of demographics, medical and psychiatric conditions, and gender dysphoria/incongruence treatment in transgender/gender diverse individuals. Biol Sex Differ. (2020) 11(1):55. 10.1186/s13293-020-00332-5 33023634 PMC7539507

[11] Mezza F Mezzalira S Pizzo R Maldonato NM Bochicchio V Scandurra C. Minority stress and mental health in European transgender and gender diverse people: a systematic review of quantitative studies. Clin Psychol Rev. (2024) 107:102358. 10.1016/j.cpr.2023.102358 37995435

[12] Marconi M Ruocco A Ristori J Bonadonna S Pivonello R Meriggiola MC. Stratified analysis of health and gender-affirming care among Italian transgender and gender diverse adults. J Endocrinol Invest. (2025) 48(6):1457–71. 10.1007/s40618-025-02547-y 39954195

[13] Marconi M Pagano MT Ristori J Bonadonna S Pivonello R Meriggiola MC. Sociodemographic profile, health-related behaviours and experiences of healthcare access in Italian transgender and gender diverse adult population. J Endocrinol Invest. (2024) 47(11):2851–64. 10.1007/s40618-024-02362-x 38733428

[14] Leppel K. Transgender men and women in 2015: employed, unemployed, or not in the labor force. J Homosex. (2021) 68(2):203–29. 10.1080/00918369.2019.1648081 31403900

[15] Meyer IH. Prejudice, social stress, and mental health in lesbian, gay, and bisexual populations: conceptual issues and research evidence. Psychol Bull. (2003) 129(5):674–97. 10.1037/0033-2909.129.5.674 12956539 PMC2072932

[16] Pellicane MJ Ciesla JA. Associations between minority stress, depression, and suicidal ideation and attempts in transgender and gender diverse (TGD) individuals: systematic review and meta-analysis. Clin Psychol Rev. (2022) 91:102113. 10.1016/j.cpr.2021.102113 34973649

[17] Puckett JA Dyar C Maroney MR Mustanski B Newcomb ME. Daily experiences of minority stress and mental health in transgender and gender-diverse individuals. J Psychopathol Clin Sci. (2023) 132(3):340–50. 10.1037/abn0000814 36913272 PMC10159909

[18] Kaplan RL El Khoury C Wehbe S Lize N Mokhbat J. Pilot results from the first HIV/AIDS intervention among transgender women in the Middle East: gender affirmation and social support from within trans communities in Beirut, Lebanon. AIDS Res Hum Retroviruses. (2020) 36(6):501–12. 10.1089/AID.2019.0203 31914788 PMC7262636

[19] Kaplan RL McGowan J Wagner GJ. HIV Prevalence and demographic determinants of condomless receptive anal intercourse among trans feminine individuals in Beirut, Lebanon. J Int AIDS Soc. (2016) 19(3S2):20787. 10.7448/IAS.19.3.20787 27431468 PMC4949314

[20] Abboud S Seal DW Pachankis JE Khoshnood K Khouri D Fouad FM. Experiences of stigma, mental health, and coping strategies in Lebanon among Lebanese and displaced Syrian men who have sex with men: a qualitative study. Soc Sci Med. (2023) 335:116248. 10.1016/j.socscimed.2023.116248 37742387

[21] Kohnepoushi P Nikouei M Cheraghi M Hasanabadi P Rahmani H Moradi M. Prevalence of suicidal thoughts and attempts in the transgender population of the world: a systematic review and meta-analysis. Ann Gen Psychiatry. (2023) 22(1):28. 10.1186/s12991-023-00460-3 37543583 PMC10403892

[22] Baral SD Poteat T Strömdahl S Wirtz AL Guadamuz TE Beyrer C. Worldwide burden of HIV in transgender women: a systematic review and meta-analysis. Lancet Infect Dis. (2013) 13(3):214–22. 10.1016/S1473-3099(12)70315-8 23260128

[23] Connolly D Gilchrist G. Prevalence and correlates of substance use among transgender adults: a systematic review. Addict Behav. (2020) 111:106544. 10.1016/j.addbeh.2020.106544 32717497

[24] Naal H Abboud S Harfoush O Mahmoud H. Examining the attitudes and behaviors of health-care providers toward LGBT patients in Lebanon. J Homosex. (2020) 67(13):1902–19. 10.1080/00918369.2019.1616431 31125288

[25] Abdallah W Najib B Khalil K Atallah D. The gender affirming surgery in a conservative religious country: the Lebanese experience. Future Sci OA. (2023) 9(5):FSO854. 10.2144/fsoa-2022-0039 37180604 PMC10167714

[26] Zucker KJ. Epidemiology of gender dysphoria and transgender identity. Sex Health. (2017) 14(5):404. 10.1071/SH17067 28838353

[27] Coleman E Radix AE Bouman WP Brown GR De Vries ALC Deutsch MB. Standards of care for the health of transgender and gender diverse people, version 8. Int J Transgender Health. (2022) 23(1):S1–259. 10.1080/26895269.2022.2100644 PMC9553112 36238954

[28] Tordoff DM Wanta JW Collin A Stepney C Inwards-Breland DJ Ahrens K. Mental health outcomes in transgender and nonbinary youths receiving gender-affirming care. JAMA Netw Open. (2022) 5(2):e220978. 10.1001/jamanetworkopen.2022.0978 35212746 PMC8881768

